# PD-L1/PD-1 expression and tumor-infiltrating lymphocytes in conjunctival melanoma

**DOI:** 10.18632/oncotarget.18039

**Published:** 2017-05-20

**Authors:** Jinfeng Cao, Niels J. Brouwer, Kate E. Richards, Marina Marinkovic, Sjoerd van Duinen, Daan Hurkmans, Els M.E. Verdegaal, Ekaterina S. Jordanova, Martine J. Jager

**Affiliations:** ^1^ Department of Ophthalmology, Leiden University Medical Center, Leiden, The Netherlands; ^2^ Department of Ophthalmology, The Second Hospital of Jilin University, Changchun, China; ^3^ Department of Obstetrics and Gynaecology, Center for Gynaecological Oncology Amsterdam (CGOA), VU University Medical Center, Amsterdam, The Netherlands; ^4^ Department of Pathology, Leiden University Medical Center, Leiden, The Netherlands; ^5^ Department of Medical Oncology, Leiden University Medical Center, Leiden, The Netherlands

**Keywords:** conjunctival melanoma, PD-L1, PD-1, CTL, immunotherapy

## Abstract

Conjunctival melanoma (CM) is an infrequent but potentially lethal malignancy, with limited therapeutic options for metastases. Recent inhibitors of the interaction of programmed cell death protein 1 (PD-1) and its ligand PD-L1 are associated with good clinical responses in many malignancies. To investigate the therapeutic potential of targeting the PD-1/PD-L1 axis in CM, we analyzed the expression of PD-1 and PD-L1 and the density of various types of tumor-infiltrating lymphocytes (TILs) in primary CM (*n* = 27), using immunofluorescence staining. Results were compared with clinical parameters and outcome. Flow cytometry was exploited to determine the PD-L1 and PD-1 protein expression in conjunctival and cutaneous melanoma cell lines. PD-L1 expression was identified on tumor cells in five (19%) primary CM and on stromal cells (mainly CD68^+^CD163^+^ M2 macrophages) in 16 (59%) cases. PD-L1 expression on tumor cells was associated with the presence of distant metastases and a worse melanoma-related survival. PD-1 expression was seen in 17 (63%) cases, all of which were T2 stage tumors. Small tumors had a higher density of TILs than large tumors. The density of TILs was not correlated with survival, tumoral/stromal PD-L1 or PD-1 expression. *In vitro* results showed that most CM and cutaneous melanoma cell lines do not constitutively express PD-L1. However, expression could be upregulated after interferon gamma stimulation. Our findings suggest that blocking the PD-1/PD-L1 axis should be evaluated as a treatment for CM.

## INTRODUCTION

Conjunctival melanoma (CM) is a rare ocular malignancy, accounting for 5% of all ocular melanoma [1]. CM is a subtype of mucosal melanoma, which is possibly associated with ultraviolet light exposure [2]. The incidence in Caucasians has risen in the last few decades to 0.8/million [3]. CM arises from melanocytes in the conjunctiva, often presenting as a brownish lesion on the eye. Most frequently, CM develops in primary acquired melanosis (PAM) (up to 74%), and less frequently in a nevus (7%) or de novo (19%) [4]. Treatment of primary CM generally consists of wide local excision followed by adjuvant treatment with either cryotherapy, brachytherapy, or topical chemotherapy [5]. Radical surgical procedures like exenteration are reserved for the most advanced stages [5]. The local recurrence rate is high, and may reach 60% in patients after 5 years, with a 5-year melanoma-related death rate of 14% [6]. Treatment options for metastasis of conjunctival melanoma are currently limited.

Recently, immunotherapies aiming at immune checkpoint pathways, such as cytotoxic T lymphocyte antigen 4 (CTLA-4) and programmed death 1 (PD-1), have been successfully exploited in the treatment of metastases of different malignancies and have led to long-lasting clinical responses [7]. Both CTLA-4 and PD-1 are upregulated on the surface of activated T cells and can bind to their respective ligands: CTLA-4 binds to B7 on antigen-presenting cells (APCs), and subsequently prevents the delivery of co-stimulatory signals and therefore the activation of T cells. PD-1 on T cells binds to the programmed death ligand 1 (PD-L1), a major PD-1 ligand which is present on the cell surface of tumor cells and macrophages, and functionally impairs the activated T cell, thereby preventing it from mounting an effective immune response against tumor antigens. Monoclonal antibodies that inhibit the interaction between PD-1 and PD-L1 block this inhibitory function and have led to improved survival in patients with metastases of cutaneous melanoma, colorectal cancer and non-small cell lung cancer [8–10].

CM in many ways resembles cutaneous melanoma, suggesting that patients with CM metastases might also benefit from treatment with anti-PD-1/PD-L1 agents. PD-L1 expression determined by immunohistochemistry (IHC) on tumor cells is thought to be a potential biomarker predicting the sensitivity of anti-PD-1/PD-L1 treatment [11–13]. Whether blocking the PD-1/PD-L1 axis will be an effective therapy for CM may therefore depend on the PD-1/PD-L1 expression status of CM. To further elucidate the role of the PD-1/PD-L1 axis in CM, and its potential interrelationship with the tumor microenvironment, we studied PD-1/PD-L1 expression and the presence of tumor-infiltrating lymphocytes (TILs) in a cohort of primary CM, and compared expression and (co)localization of these factors to clinical and histological characteristics.

## RESULTS

### Patient characteristics

We studied primary CM from 27 patients who had been treated at the LUMC between 1996 and 2014 (Table 1). Fifteen (56%) patients were female, and 14 (52%) were over 60 years old. The epibulbar localization (*n* = 20) is comprised of limbal (*n* = 16) and bulbar conjunctiva (*n* = 4). The non-epibulbar localization (*n* = 7) includes tarsal, forniceal and caruncular conjunctiva. The clinical TNM stage was T1 in 20 (74%) and T2 in 7 (26%) cases. Two (7%) of the patients underwent surgical excision alone as primary treatment, three (11%) excision with cryotherapy, one (4%) excision and mitomycin C, 16 (59%) excision and subsequent brachytherapy, one (4%) external beam radiation, and four (15%) were treated by exenteration. The median follow-up time was 46 months (range 3–247 months). Eleven (41%) cases developed local recurrences. At the end of the follow-up period, four patients had died from CM metastases, two from unknown diseases without any signs of metastases, and 21 patients were alive.

**Table 1:** Clinicopathological characteristics and correlation with PD-L1 and PD-1 expression

Characteristic		All cases	Tumoral PDL1			Stromal PDL1			Stromal PD1		
			Negative	Positive		Negative	Positive		Negative	Positive	
		Cases (%)	Cases (%)	Cases (%)	p	Cases (%)	Cases (%)	p	Cases (%)	Cases (%)	p
Overall		27 (100)	22 (81)	5 (19)		11 (41)	16 (59)		10 (37)	17 (63)	
Sex											
	Male	12 (44)	10 (45)	2 (40)	1.00*	4 (36)	8 (50)	0.70*	4 (40)	8 (47)	1.00*
	Female	15 (56)	12 (55)	3 (60)		7 (64)	8 (50)		6 (60)	9 (53)	
Age at diagnosis											
	Age ≤60 year	13 (48)	12 (55)	1 (20)	0.33*	8 (73)	5 (31)	0.03**	5 (50)	8 (47)	1.00*
	Age >60 year	14 (52)	10 (45)	4 (80)		3 (27)	11 (69)		5 (50)	9 (53)	
Tumor size, thickness		N=23	N=20	N=3		N=10	N=13		N=9	N=14	
	Median [range], mm	1.0 [0.1-16.0]	0.9 [0.2-5.0]	6.0 [0.1-16.0]	0.40^&^	0.8 [0.2-5.0]	1.4 [0.1-16.0]	0.65^&^	0.6 [0.2-5.0]	1.2 [0.1-16.0]	0.56^&^
Tumor size, LBD		N=24	N=19	N=5		N=10	N=14		N=9	N=15	
	Median [range], mm	9.5 [2.0-30.0]	10.0 [2.0-30.0]	6.0 [5.0-20.0]	0.89^&^	9.5 [2.0-12.0]	10.0 [2.0-30.0]	0.44^&^	7.0 [2.0-15.0]	10.0 [2.0-30.0]	0.48^&^
Location											
	Epibulbar	20 (74)	17 (77)	3 (60)	0.58*	9 (82)	11 (69)	0.66*	10 (100)	10 (59)	0.03*
	Non-epibulbar	7 (26)	5 (23)	2 (40)		2 (18)	5 (31)		0 (0)	7 (41)	
cTNM**											
	T1	20 (74)	17 (77)	3 (60)	0.58*	10 (82)	11 (69)	0.66*	10 (100)	10 (59)	0.03*
	T2	7 (26)	5 (23)	2 (40)		2 (18)	5 (31)		0 (0)	7 (41)	
Local recurrence											
	No	16 (59)	13 (59)	3 (60)	1.00*	6 (55)	10 (63)	0.71*	8 (80)	8 (47)	0.12*
	Yes	11 (41)	9 (41)	2 (40)		5 (45)	6 (38)		2 (20)	9 (53)	
Distant metastasis											
	No	23 (85)	21 (95)	2 (40)	0.01*	11 (100)	12 (75)	0.12*	10 (100)	13 (76)	0.27*
	Yes	4 (15)	1 (5)	3 (60)		0 (0)	4 (25)		0 (0)	4 (24)	

LBD = largest basal diameter. cTNM = clinical TNM stage, based on the first occurring conjunctival melanoma. *P* value calculation: * = Fischer exact test; ** = Pearson's chi-square; ^&^ = Mann Whitney U test. Italic *P* values are ≤ 0.05.

### Expression of PD-L1/PD-1 and TILs in CM

We determined PD-L1 expression on sections of 27 CM that were co-stained with HMB45/MART-1 antibody. The combination allowed us to distinguish between PD-L1 expressing tumor cells versus non-tumor cells. The PD-L1 positive non-tumor cells were mainly comprised of macrophages, similar to what has been described previously [14].

**Figure 1:**
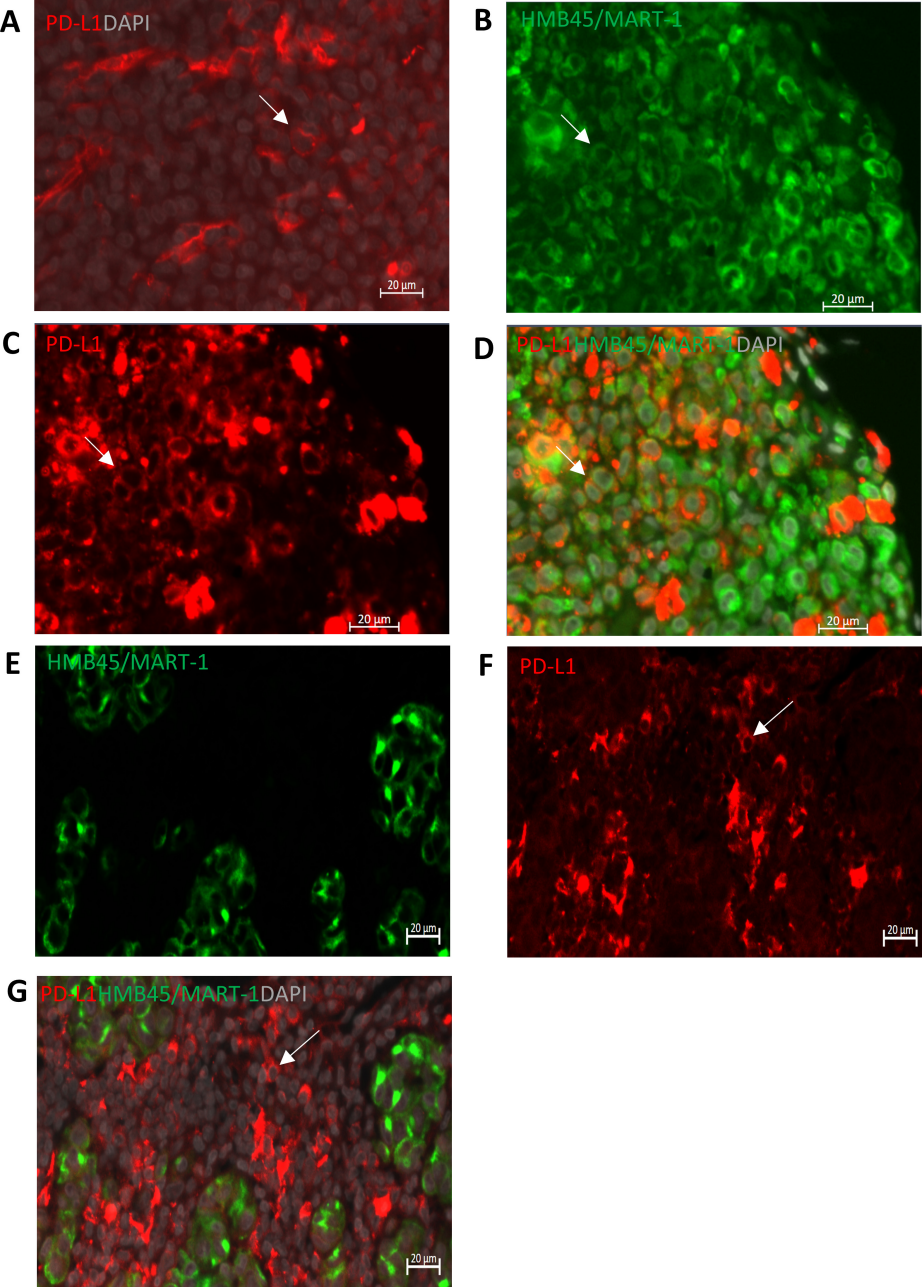
PD-L1 expression in primary CM as determined by IF analysis (**A**) Positive membranous PD-L1 (red) staining in the positive control, human tonsil tissue. (**B**–**D**) Representative images of HMB45/MART-1 (B, green, cytoplasmic/membranous), PD-L1 (**C**, red, membranous) and double staining (D) with DAPI (grey), show that PD-L1 is expressed on CM cells. (**E**–**G**) PD-L1 is expressed on HMB45/Mart-1 negative stromal cells. Scale bar is 20μm. White arrows indicate the positive cells.

**Figure 2:**
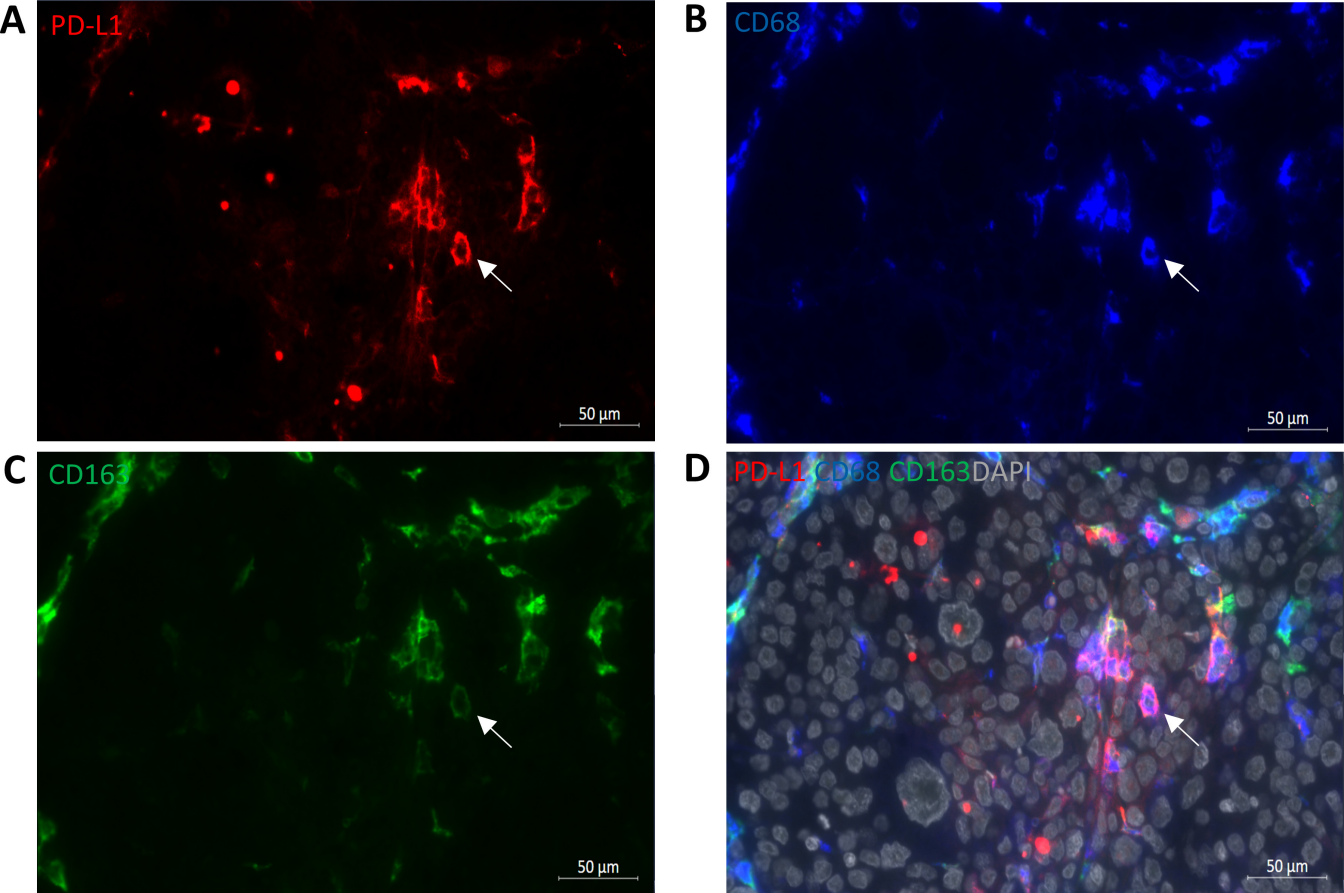
PD-L1 positive stromal cells are primarily CD68^+^CD163^+^ macrophages (**A**) PD-L1 (red, membranous), (**B**) CD68 (blue, cytoplasmic/membranous), (**C**) CD163 (green, cytoplasm/membrane) and merged image (**D**) with DAPI (grey) show that PD-L1 positive stromal cells are also CD68^+^CD163^+^ positive cells. White arrow indicates the positive staining. Scale bar is 50 μm.

Using a cut-off value of 5%, tumoral and stromal PD-L1 membranous expression was identified in five (19%) and 16 (59%) CM sections, respectively, as illustrated in Figure 1 and Table 1. One tumor showed 30% tumoral PD-L1 expression, while the other four cases had between 5–10% of the tumor cells expressing PD-L1. Published cut-off points used to define PD-L1 positivity vary from 1% to 50% [13]. As only one sample had sporadic PD-L1 positive tumor cells (1% to 5%) in our cohort, we decided to use 5% as cut-off point for comparisons. PD-L1 expression in stroma was seen more often in patients over 60 (*p* = 0.03), while positive PD-L1 staining in tumor areas was associated with the development of distant metastases (*p* = 0.01). Kaplan-Meier analysis and log rank testing similarly showed that PD-L1 positive staining in the tumor was associated with a worse melanoma-related survival (*p* = 0.045; Figure 4). Furthermore, to better understand the nature of PD-L1 positive cells in stroma, we stained sections from seven CM that contained PD-L1 positive stromal cells with anti-PD-L1, CD68 and CD163 antibodies. We observed that PD-L1 positive stromal cells were mainly CD68^+^CD163^+^ cells (Figure 2).

**Figure 3:**
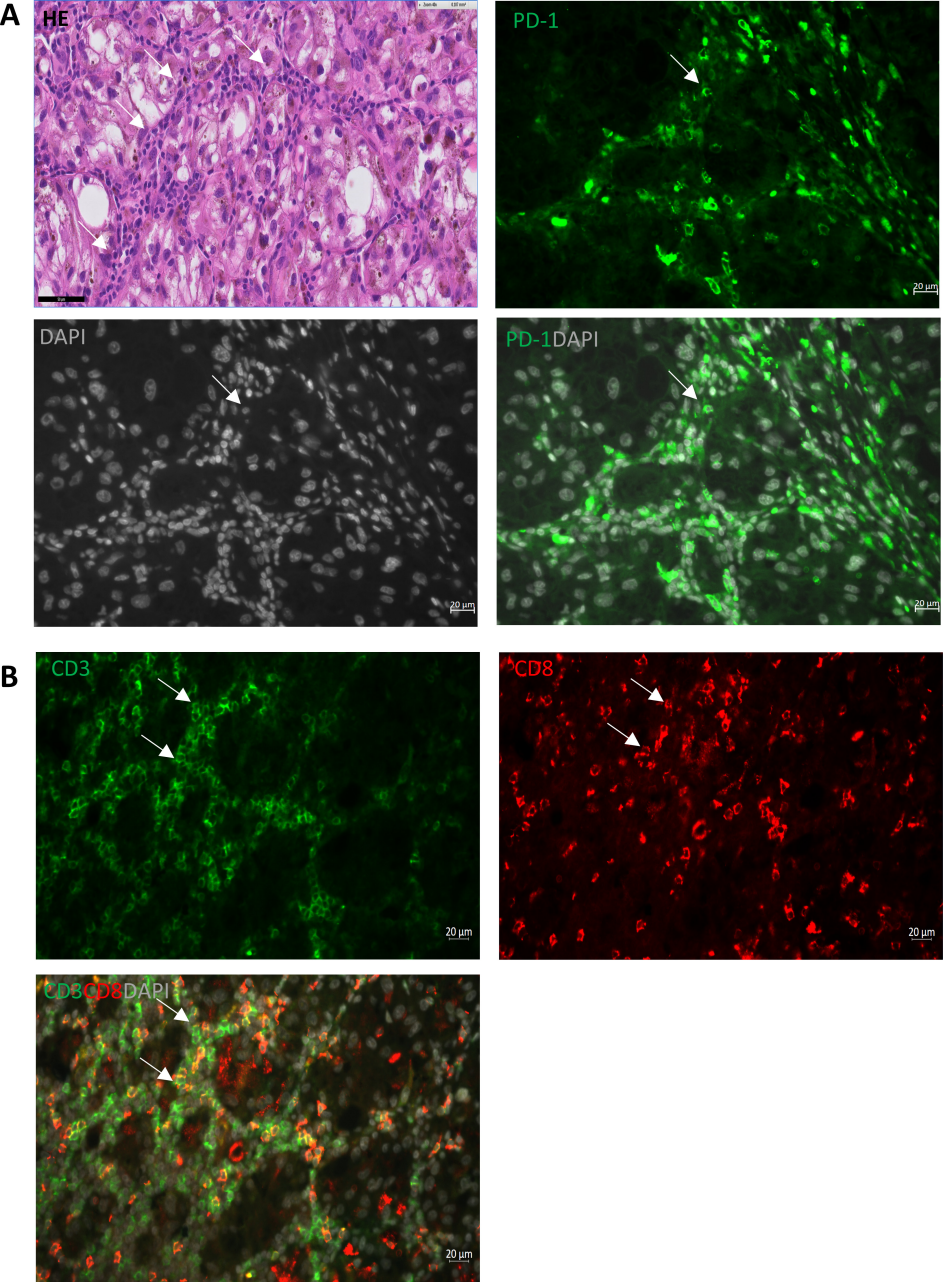
PD-1 expression in CM Representative immunohistological staining shows that: (**A**) PD-1 (green, membrane) is expressed on stromal cells surrounding the primary tumor areas (white arrows); (**B**) staining of CD3 (green) and CD8 (red) demonstrates these stromal cells are T cells (white arrows). Scale bar of IF is 20 μm, and of HE is 50 μm.

**Figure 4:**
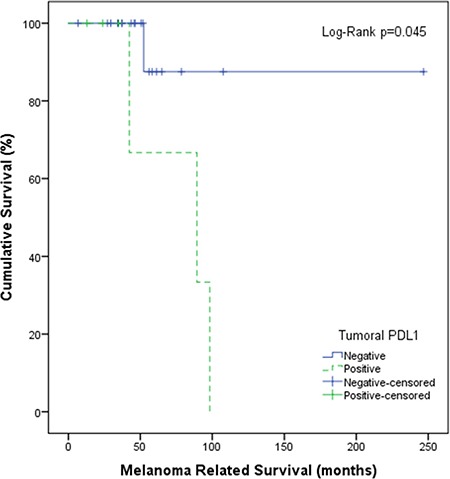
Survival analysis according to PD-L1 status in CM Kaplan-Meier plot shows disease-specific survival of patients with PD-L1-positive tumors (green, dotted) and negative tumors (blue, continuous) (cut-off at 5%). *P*-value has been calculated using the log-rank test.

PD-1 expression was localized on the membrane of T cells (Figure 3), and was seen in 17 (63%) CM samples. All tumors at T2 stage were PD-1 positive (*p* = 0.03). Absence of PD-1 tended to correlate with less local recurrence (*p* = 0.12). A prior study on cutaneous melanoma showed that those melanomas often harbor intrinsically PD-1-positive tumor cell subpopulations [15]; however, we did not find positive PD-1 staining on the tumor cells themselves.

**Figure 5:**
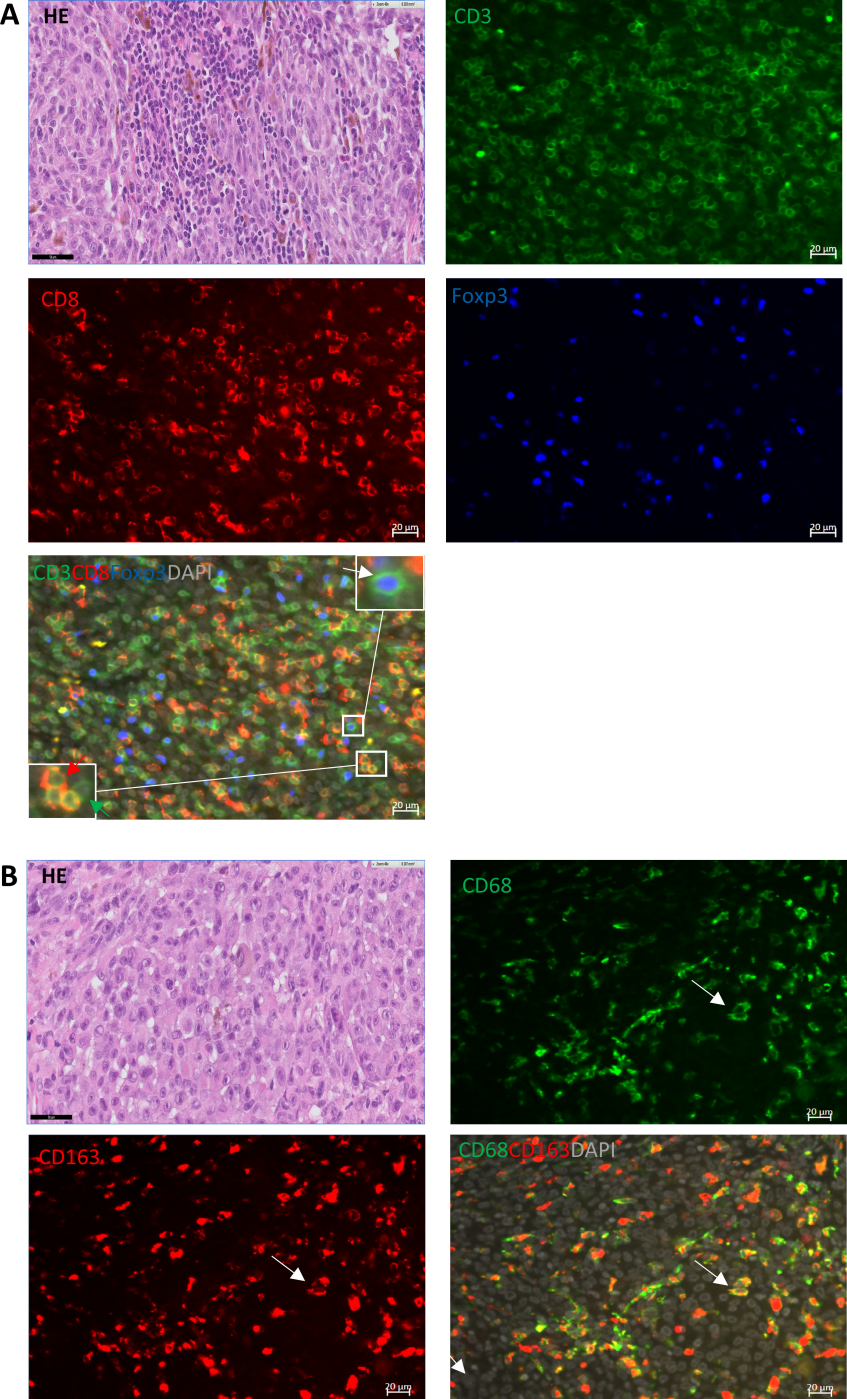
T cell and macrophage subset analysis in the tumor area of CM (**A**) HE, CD3 (green, membrane), CD8 (red, membrane), Foxp3 (blue, nucleus) and the merged image; the combination of nuclear blue Foxp3 and surface green CD3 staining (white arrow) indicates the presence of CD3^+^CD8^-^Foxp3^+^ T cells. The green arrow indicates a CD3^+^CD8^-^Foxp3^-^ T cell, and the red arrow points at CD3^+^CD8^+^ T cells. (**B**) HE, CD68 (green, cytoplasm/membrane), CD163 (red, cytoplasm/membrane) and merged image shows double-positive M2 macrophages cells. Scale bar of IF is 20 μm, and of HE is 50 μm.

In order to see whether specific types of infiltrating leukocytes contributed to PD-L1 and PD-1 expression on tumor cells, we determined the presence of different subsets of T cells and myeloid cells in the same CM, by performing immunofluorescence (IF) staining according to previously described techniques [16]: we measured the numbers of CD3, CD3^+^CD8^+^, CD3^+^CD8^-^, CD3^+^CD8^-^Foxp3^+^ and CD3^+^CD8^-^Foxp3^-^ T cells, and CD68 (macrophages) and CD68^+^CD163^+^ (M2 macrophages) within tumor areas of 26 primary CM sections. Figure 5 shows an example of a tumor with a high number of infiltrating lymphocytes. In general, all tumors presented a wide variety of different types of TILs (Table 2). T2 tumors showed less infiltration with CD3^+^CD8^-^ and CD3^+^CD8^-^Foxp3^-^ positive cells than non-T2 tumors (*p* = 0.048 and 0.02, respectively, Table 2). Although the CD3^+^CD8^-^Foxp3^+^ regulatory T cells may function as suppressors of effector T cells, Spearman rank analysis showed significantly positive associations between the density of CD3^+^CD8^-^Foxp3^+^ and of CD3, CD3^+^CD8^+^, CD3^+^CD8^-^ as well as of CD3^+^CD8^-^Foxp3^-^ T cells (Supplementary Table 1). The different subsets of T cells frequently co-infiltrate CM. As tumor thickness is a known prognostic risk factor for CM [17], we correlated the density of TILs with tumor thickness, and observed that thicker tumors had less CD3^+^CD8^+^ T cells (*p* = 0.03) and tumor-infiltrating M2 macrophages (*p* = 0.02; Table 3). Tumors with larger basal diameters contained fewer infiltrating CD3 (*p* = 0.01), CD3^+^CD8^+^ (*p* = 0.02), CD3^+^CD8^-^ (*p* = 0.01), CD3^+^CD8^-^Foxp3^-^ (*p* = 0.02) and CD3^+^CD8^-^Foxp3^+^(*p* = 0.03) T cells within their tumor areas than tumors with smaller basal diameters (Table 3). The density of all types of TILs mentioned above was not correlated with tumoral/stromal PD-L1 expression (*p* > 0.05) or with melanoma-related survival. IF staining of CD68 and CD68^+^CD163^+^ showed that the majority of macrophages belong to the M2 phenotype, suggesting a potential tumor-favorable environment created by macrophages in CM. As high cytotoxic T lymphocyte (CTL)/regulatory T cell (Treg) and high M1 (CD68^+^CD163^-^)/M2 macrophage ratios have been found to be associated with improved survival in breast cancer and cutaneous melanoma, respectively [14, 18], we evaluated these ratios in our study. No significant difference in survival or association with clinical parameters was observed (*p* > 0.05). However, higher CTL/Treg ratio tended to correlate with local recurrence (*p* = 0.13). Correlation coefficients are shown in Table 3 and Supplementary Table 1.

**Table 2:** Baseline clinicopathological characteristics and correlation with tumor-infiltrating lymphocytes

Categorical variables		Cases (%)	CD3		CD3^+^CD8^+^		CD3^+^CD8^-^		CD3^+^CD8^-^Foxp3^-^		CD3^+^CD8^-^Foxp3^+^		CD68		CD68^+^CD163^+^	
		Median [range]	p	Median [range]	p	Median [range]	p	Median [range]	p	Median [range]	p	Median [range]	p	Median [range]	p
Overall		26 (100)	151 [71–637]		68 [26–335]		70 [26–314]		44 [13–202]		30 [10–124]		59 [19–248]		39 [8–220]	
Sex																
	Male	11 (42)	130 [71–637]	0.33	65 [26–335]	0.51	65[26–302]	0.33	39 [13–202]	0.22	32 [11–100]	0.72	39 [19–248]	0.10	30 [8–220]	0.54
	Female	15 (58)	159 [85–617]		80 [43–303]		83 [30–314]		53 [13–190]		26[10–124]		63 [22–189]		41 [11–161]	
Age at diagnosis, yr																
	≤60	12 (46)	137 [85–299]	0.86	66 [28–170]	0.86	73 [26–173]	0.98	48 [15–100]	1.00	30 [11–80]	0.86	40 [19–76]	0.08	29 [8–59]	0.09
	>60	14 (54)	158 [71–637]		74 [26–335]		69 [30–314]		42 [13–202]		30 [10–124]		63 [19–248]		43 [18–220]	
Location																
	Epibulbar	19 (73)	171 [71–637]	0.08	82 [26–335]	0.15	83 [26–314]	0.048	53 [15–202]	0.02	34 [10–124]	0.53	63 [19–248]	0.12	41 [8–220]	0.23
	Non-epibulbar	7 (27)	121 [110–144]		58 [50–80]		63 [30–76]		35 [13–51]		25 [17–48]		47 [19–63]		22 [11–59]	
cTNM																
	T1	19 (73)	171 [71–637]	0.08	82 [26–335]	0.15	83 [26–314]	0.048	53 [15–202]	0.02	34 [10–124]	0.53	63 [19–248]	0.12	41 [8–220]	0.23
	T2	7 (27)	121 [110–144]		58 [50–80]		63 [30–76]		35 [13–51]		25 [17–48]		47 [19–63]		22 [11–59]	
Local recurrence																
	No	15 (58)	125[71–617]	0.61	58 [26–303]	0.28	68 [30–314]	0.88	42 [13–189]	0.88	27 [10–124]	0.84	57 [19–189]	1.00	41 [8–161]	1.00
	Yes	11 (42)	161 [85–637]		74 [43–335]		79 [26–302]		45 [13–202]		32 [11–100]		63 [22–248]		38 [11–220]	
Distant metastasis																
	No	22 (85)	151 [71–637]	0.86	66 [26–335]	0.76	70 [26–314]	0.52	46 [15–202]	0.20	27 [10–124]	0.76	63 [19–248]	0.17	43 [8–220]	0.28
	Yes	4 (15)	142 [110–207]		77 [62–82]		70 [30–133]		27 [13–92]		39 [17–48]		40 [27–47]		26 [19–39]	

cTNM = clinical TNM stage, based on the first occurring conjunctival melanoma. *P* values were calculated by Mann Whitney *U* test. *P* ≤ 0.05 are in italics.

**Table 3:** Correlation between different types of infiltrating immune cells and tumor size

	Tumor thickness		Tumor LBD	
	*r*	*p*	*r*	*p*
CD3	–0.40	0.06	–0.56	*0.01*
CD3^+^CD8^+^	–0.45	*0.03*	–0.50	*0.02*
CD3^+^CD8^-^	–0.36	0.09	–0.54	*0.01*
CD3^+^CD8^-^Foxp3^-^	–0.39	0.07	–0.50	*0.02*
CD3^+^CD8^-^Foxp3^+^	–0.32	0.19	–0.46	*0.03*
CD68	–0.38	0.07	–0.26	0.24
CD68^+^CD163^+^	–0.49	*0.02*	–0.23	0.30
Tumor thickness	–	–	0.65	*0.001*

*r* = two-tailed Spearman correlation coefficient, with 26 observations. LBD = largest basal diameter. *P* ≤ 0.05 are in italics.

### Human CM cell lines express various levels of PD-L1

**Figure 6:**
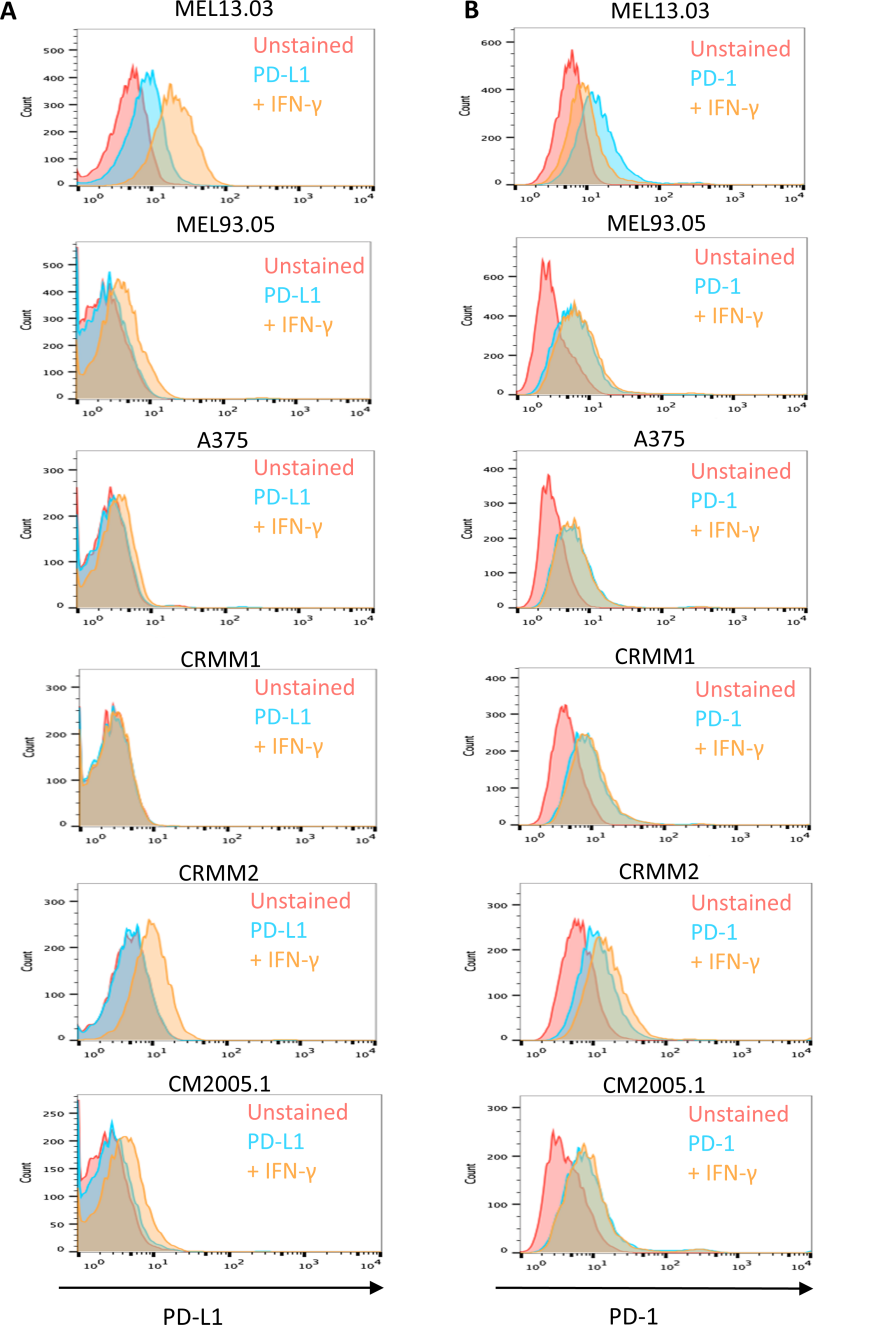
Cutaneous (MEL13.03, MEL93.05 and A375) and conjunctival melanoma (CRMM1, CRMM2 and CM2005.1) cell lines express various levels of PD-L1 and PD-1 MEL13.03 is the positive control cell line for both PD-L1 and PD-1. Representative histograms show (**A**) PD-L1 and PD-1 (**B**) expression in cell lines with or without IFN-γ (100 IU/ml) exposure for 48 h. Pink, blue and brown shaded histograms represent unstained, PD-L1 (PD-1) staining, and the effect of IFN-γ stimulation on PD-L1 and PD-1, respectively.

Infiltration lymphocytes may be a source of interferon gamma (IFN-γ), which has been reported to enhance PD-L1 and PD-1 expression [19, 20]. To examine how PD-L1 and PD-1 are expressed on the cell surface of CM cell lines, and to determine whether expression is sensitive to environmental cytokines, we performed flow cytometry on three human cutaneous melanoma and three CM cell lines. The cutaneous melanoma cell line MEL13.03 served as PD-L1 and PD-1 positive control. Figure 6 shows that compared to MEL13.03, the other five cell lines were PD-L1 negative, while only one other cell line, CRMM2, expressed PD-1. Next, to mimic the immune environment *in vivo*, we stimulated these cells with IFN-γ. As a control, we determined the upregulation of IFN-γ pathway by analyzing HLA Class I expression, using an anti-HLA class I antibody (Supplementary Figure 1). HLA Class I expression of all cell lines was upregulated upon IFN-γ stimulation. After 48 h incubation with IFN-γ, PD-L1 expression was upregulated at different levels on two of the three cutaneous melanoma cell lines (MEL13.03, MEL93.05) and on two of the three CM cell lines tested (CRMM2 and CM2005.1), while PD-1 was only slightly increased on one cell line (CRMM2) (Figure 6).

## DISCUSSION

Immunotherapies that work through inhibiting the PD-L1/PD-1 axis have been successful in inducing clinical responses in patients with different malignancies, including cutaneous melanoma, non-small cell lung cancer, and bladder cancer [21–23]. However, there are no data yet on the expression of immune checkpoint molecules in CM, a rare malignancy. As far as we know, one ongoing clinical trial testing the efficacy and safety of Ipilimumab in metastatic melanoma patients is currently recruiting CM patients (NCT01355120). Very recently, a CM patient with a breast metastasis was successfully treated with Nivolumab, a monoclonal antibody directly against PD-1 [24]. However, the PD-L1 expression of the primary or metastatic tumor of the patient was not described. Although the accuracy and reproducibility of PD-L1 staining is disputable, and the clinical responses may occur in PD-L1 negative tumors and not all PD-L1 positive tumors respond [23], immunostaining is the best attempt to spredict the potential of immune-based therapies [25]. Since most CM are small and heterogenous, and some are pigmented, we decided to use the anti-PD-L1 SP142 clone as it has been shown to work in IF staining on paraffin-embedded sections [14, 23]. In addition, it has recently been approved by the FDA as a complementary diagnostic to help make treatment decisions for the use of Atezolizumab in patients with non-small cell lung cancer. We determined whether PD-L1 expression was located on tumor cells or cells of the tumor microenvironment by simultaneous staining with a melanoma marker.

PD-L1 expression is a potential biomarker for prognosis in different types of cancer [26–29]. Expression of PD-L1 has been investigated in varies malignancies with most researchers using either a 1% or 5% cut-off for positivity [30]. Cytoplasmic staining of PD-L1 has often been neglected because the significance of intracellular expression of PD-L1 remains unclear and does not seem functional [31]. In the present study of a human CM cohort, we found that 19% of the tumors expressed PD-L1 (cut-off 5%), and that this expression was correlated with the presence of distant metastases and a worse melanoma-related survival. The incidence of tumor PD-L1 expression is lower than cutaneous melanoma, as reported previously [32]. However, our finding should be interpreted with caution as our cohort has a limited size. More patients are needed for further analysis of the prognostic value of PD-L1 expression in CM in order to confirm our findings. Although one study shows positive PD-L1 expression in 13% (3/23) of mucosal malignant melanoma of head and neck [33], another study [34] did not find any clinical response by application of PD-1 inhibitors in a group of patients with advanced recurrent mucosal melanoma of head and neck. However, the cohort is rather small (*n* = 5).

Not only expression of PD-L1 on tumor cells may be important, also PD-L1 expressed by myeloid cells in the tumor microenvironment may play an essential role in suppression of the host's immune response, even when the malignant cells lack PD-L1 [14, 35]. Stromal PD-L1 expression can predict poor prognosis in adult T-cell leukemia or lymphoma and gastric carcinoma [9, 29]. Here, we observe that 59% of CM contained PD-L1 positive stromal cells, but expression did not correlate with survival. The PD-L1 positive stromal cells were mainly comprised of CD68^+^CD163^+^ M2 macrophages, similar to what has been described previously [14]. *In vitro* experiment showed that all CM cell lines expressed low levels of membranous PD-L1, and a variable but clear increase of PD-L1 expression was seen in two out of three CM cell lines following IFN-γ stimulation. These findings suggest that in CM, initially PD-L1 negative or weakly positive tumors may display enhanced PD-L1 expression after exposure to IFN-γ produced by TILs.

Cancer exploits multiple mechanisms to avoid antitumor immune responses. Based on the “cancer immunogram” depicted by Blank, et al. [36], the general immune status and immune cell infiltration needs to be addressed to facilitate the understanding of immune-based treatments. Unlike another type of ocular melanoma, uveal melanoma (UM), the immunology of CM has hardly been studied. Although the unique conjunctiva-associated lymphoid tissue (CALT) system in conjunctiva especially contains B lymphocytes [37], we mainly focus on T lymphocytes because the PD-L1/PD-1 axis inactivates T-cell function. When we compare expression with the cell counts of TILs in UM, using the same antibodies and techniques as in our prior study on UM, we notice that CM contain higher densities of CD4 (CD3^+^CD8^-^), CD4 helper (CD3^+^CD8^-^Foxp3^-^) and Foxp3 (CD3^+^CD8^-^Foxp3^+^) cells than UM. However, the densities of CD8 (CD3^+^CD8^+^), CD68 and CD68CD163 cells were lower than those in UM. Compared to one study of cutaneous melanoma metastasis [38], the density of CD3 and CD68CD163 was similar, with a higher density of CD4 and Foxp3, and lower density of CD8 cells. Some studies have shown that PD-L1 expression inversely correlates with TILs [32, 39]. We find no association between tumoral or stromal PD-L1 positivity and the density of TILs. However, the density of TILs was inversely correlated with tumor size, with larger tumors containing fewer immune cells, suggesting that in the absence of infiltrating immune cells, including cytotoxic T cells, the tumor could grow unrestrained.

A major limitation of the present study is the small size of the cohort, coming from a single institute, due to the rarity of CM. We need more patients and tumor material, especially metastases, to carry out further studies and draw solid conclusions. In addition, we should be aware that CM samples are generally quite small, and that a representative section accounts for a small volume of tumor, and may not represent the PD-L1 expression of the whole tumor, as it is known that PD-L1 expression may be quite heterogeneous [35].

In general, we provide a comprehensive view of PD-L1 and PD-1 protein expression, and immune infiltration status in CM. These findings deepen our understanding of the immunology of CM. We believe that these results support the rationale of PD-L1/PD-1 checkpoint immunotherapy for patients with metastatic CM and recommend to include these patients in future immunotherapy clinical trials inhibiting the PD-L1/PD-L1 pathway.

## MATERIALS AND METHODS

### Patient data

Twenty-seven patients with histologically-proven primary CM were included in this study. All patients were seen at the Leiden University Medical Center, The Netherlands, and diagnosed with CM between 1996 and 2014. The medical files were reviewed for clinical and histopathological data. Information regarding the localisation and size of the primary tumors was obtained from the patient files, histology reports, and pre-excision color photographs. All tumors were evaluated by an experienced ophthalmic pathologist. Tumor stage was determined according to the 7^th^ edition of the AJCC TNM cancer staging manual [40]. Treatment was defined as the initial treatment applied immediately or directly after histologic confirmation of CM. Local recurrence was defined as recurrence of histologically-proven CM. Metastasis was proven by histology or imaging. Total follow up time was defined as the time from diagnosis to the last known moment of survival or death. The study adhered to the tenets of the declaration of Helsinki, and the institutional Medical Ethical Committee of LUMC did not object to this retrospective analysis.

### Immunofluorescence staining

Formalin-fixed, paraffin-embedded blocks containing tumor material were cut in 4 μm sections, and mounted on slides. After deparaffinization with Xylene, rehydration with alcohol (100%, 90%, 80%, 70%), and Tris-EDTA (pH 9.0) heat-based antigen retrieval, the tissues were incubated with primary antibodies at 4°C overnight. On the second day, after washing with phosphate-buffered saline (PBS), the samples were incubated with AlexaFluor (Invitrogen, Breda, The Netherlands) secondary antibodies for 1 hour at room temperature, followed by washing steps. The slides were counterstained and mounted with VECTASHIELD mounting medium with DAPI (4′,6-diamidino-2-phenylindole; H-1200; Vector Laboratories, USA). Tonsil tissues were used as positive control. Incubation with 1% bovine serum albumin (BSA)/PBS instead of primary antibodies served as negative control. One tumor contained only enough material for PD-L1 and PD-1 staining, and not for additional staining. The primary antibodies are listed below: HMB45/Mart-1 (mouse, clone HMB45 + DT101 + BC199, ab732, 1:200; Abcam, UK), anti-PD-L1 (rabbit, clone SP142, 1:100; Spring Bioscience, CA, USA), anti-PD1 (goat, AF1086, 1:100; R&D Systems, UK), CD3 (rabbit, ab828, 1:100; Abcam), anti-CD8 (mouse IgG2b, 4B11, 1:75; Novocastra, Valkenswaard, The Netherlands), anti-FoxP3 (mouse IgG1, clone 236A/E7, 1:100; Abcam), anti-CD68 (mouse IgG2a, ab49777, 1:75; Abcam) and anti-CD163 (mouse IgG1, clone 10D6, 1:100; Novocastra). The secondary antibodies are in Supplementary Table 2.

### Imaging, scoring and analysis

The images of hematoxylin and eosin (HE) stained tumor sections were captured using Philips Image Management System 2.2. Images of IF staining were captured using either a Leica TCS SP8 X or Zeiss LSM 700 confocal laser scanning microscope. Depending on the tumor size, one to seven representative images at high power (250X) in different areas were randomly selected. Tumor areas were morphologically recognized by DAPI nuclear staining. Two investigators, without prior knowledge of clinicopathological data, scored membranous PD-L1 and PD1 expression. Expression of PD-L1 and PD-1 was designated as positive, when ≥ 5% of the tumor/stromal cells were positive [32, 41]. For evaluation of the number of tumor-infiltrating lymphocytes within the tumor sites, tumor regions (mm^2^) were evaluated using Leica Application Suite X or Zeiss Zen 2.1 software. Positive cells were counted manually by two observers, as previously described [16]. Results were presented as cell numbers/mm^2^.

### Cell lines

Three conjunctival melanoma cell lines (CRMM1, CRMM2 and CM2005.1) [42, 43] and three cutaneous melanoma cell lines (A375 (ATCC), and MEL93.05 and MEL13.03, established in the Department of Medical Oncology, LUMC, Leiden) were used in our experiments. To determine the expression of PD-L1 and PD-1 on the cell lines, cells were first seeded in 6-well plates. On the second day, media were refreshed or replaced with culture media containing 100 international units (IU)/ml of IFN-γ (ImmunoTools, Germany) and incubated for 48 h. Cells were subsequently prepared for fluorescence-activated cell sorting (FACS).

### Flow cytometry

Cells were incubated with the previously determined optimal dilution of mouse monoclonal PD-L1 (17-5983, APC; Bioscience), PD-1 (329935, FITC; BioLegend) or HLA class I antibodies (W6/32, 311414, Alexa Fluor 647; BioLegend). Cells were collected (10000-20000 per live gate) using the FACSCalibur cytometer (Becton Dickinson), and results were analysed using FlowJo software (V10.0.7, Flowjo LLC).

### Statistical analysis

Data were analysed with SPSS software version 23.0 (SPSS, Inc., Chigaco, IL, USA). Data were considered statistically significant if *p* ≤ 0.05. Pearson's chi square and Fisher's exact test were applied for categorical data; Mann Whitney *U* test was used for numerical data. Spearman's rank correlation analysis (two-tailed) was performed to compare correlations between different TILs and tumor size. Survival analysis was performed using Kaplan-Meier with log rank tests.

## SUPPLEMENTARY MATERIALS FIGURES AND TABLES



## Abbreviations

PD-1: programmed cell death protein 1
PD-L1: programmed death-ligand 1
CM: conjunctival melanoma
TIL: tumor-infiltrating lymphocytes
CTLA-4: cytotoxic T lymphocyte antigen 4
APCs: antigen-presenting cells
IHC: immunohistochemistry
IF: immunofluorescence
CTL: cytotoxic T lymphocyte
Treg: regulatory T cell
IFN-γ: Interferon-gamma
CALT: conjunctiva-associated lymphoid tissue
