# Supplementary material for: PD-L1/PD-1 expression and tumor-infiltrating lymphocytes in conjunctival melanoma
Source: Oncotarget. 2017 May 20;8(33):54722–34. doi: 10.18632/oncotarget.18039 (PMC5589616; doi:10.18632/oncotarget.18039)
Supplement: Supplementary file 1 [file oncotarget-08-54722-s001.pdf]

PD-L1/PD-1 expression and tumor-infiltrating lymphocytes in conjunctival melanoma

SUPPLEMENTARY MATERIALS

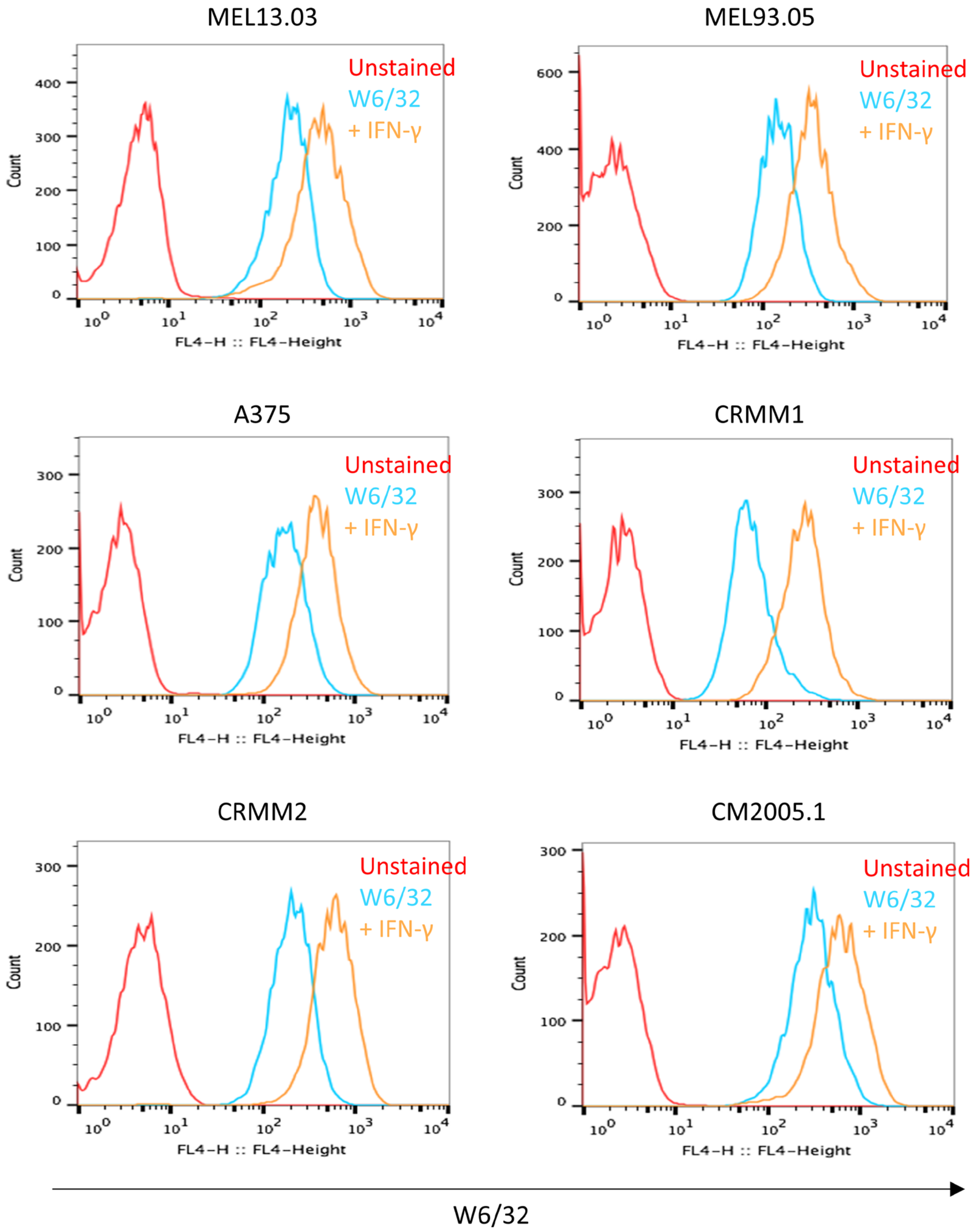

Supplementary Figure 1: The confirmation of IFN-γ effect on cutaneous (MEL13.03, MEL93.05 and A375) and conjunctival melanoma (CRMM1, CRMM2 and CM2005.1) cell lines using the anti-human HLA-A, B, C antibody (W6/32). The cells were treated with IFN-γ (100 IU/ml) for 48 h. Histograms with red, blue and brown line represent unstained, W6/32 expression, and the effect of IFN-γ stimulation on W6/32, respectively.

Supplementary Table 1: Correlation between different infiltrating immune cells (T cells and macrophages)

		$CD3^+CD8^+$	$CD3^+CD8^-$	$CD3^+CD8^-Foxp3^-$	$CD3^+CD8^-$ $Foxp3^+$	CD68	$CD68^+CD163^+$
CD3	*r*	0.84	0.95	0.88	0.84	0.63	0.48
	P	*< 0.001*	*< 0.001*	*< 0.001*	*< 0.001*	*0.001*	*0.01*
$CD3^+CD8^+$	*r*		0.69	0.63	0.57	0.59	0.46
	P		*< 0.001*	*0.001*	*0.002*	*0.001*	*0.02*
$CD3^+CD8^-$	*r*			0.93	0.84	0.53	0.38
	P			< 0.001	< 0.001	*0.005*	0.053
$CD3^+CD8^-$ $Foxp3^-$	*r*				0.65	0.51	0.34
	P				*< 0.001*	*0.01*	0.09
$CD3^+CD8^-$ $Foxp3^+$	*r*					0.49	0.46
	P					*0.01*	*0.02*
CD68	*r*						0.87
	P						*< 0.001*

r = two-tailed Spearman correlation coefficient, with 26 observations. $P \leq 0.05$ are in italics.

Supplementary Table 2: Secondary antibodies used in IF

Antibody	Specificity	Isotype	Company	Catalogue number	Dilutions
AlexaFluor 488	mouse	IgG	Life Technologies	A-11001	1:300
AlexaFluor 546	rabbit	IgG	Life Technologies	A-11010	1:300
AlexaFluor 488	goat	IgG	Life Technologies	A-11055	1:300
AlexaFluor 488	rabbit	IgG	Life Technologies	A-11034	1:300
AlexaFluor 546	mouse	IgG2b	Life Technologies	A-21143	1:300
AlexaFluor 647	mouse	IgG1	Life Technologies	A-21240	1:300
AlexaFluor 488	mouse	IgG2a	Life Technologies	A-21131	1:300
AlexaFluor 546	mouse	IgG1	Life Technologies	A-21123	1:300
AlexaFluor 647	mouse	IgG2a	Life Technologies	A-21241	1:300
AlexaFluor 488	mouse	IgG1	Life Technologies	A-21121	1:300
